# Naive T Cells in Graft Versus Host Disease and Graft Versus Leukemia: Innocent or Guilty?

**DOI:** 10.3389/fimmu.2022.893545

**Published:** 2022-06-20

**Authors:** Linde Dekker, Evy Sanders, Caroline A. Lindemans, Coco de Koning, Stefan Nierkens

**Affiliations:** ^1^ Princess Máxima Center for Pediatric Oncology, Utrecht, Netherlands; ^2^ Center for Translational Immunology, University Medical Center Utrecht, Utrecht, Netherlands

**Keywords:** allogeneic hematopoietic cell transplantation, naive T cells, graft versus host disease, graft versus leukemia, alloreactivity

## Abstract

The outcome of allogeneic hematopoietic cell transplantation (allo-HCT) largely depends on the development and management of graft-versus-host disease (GvHD), infections, and the occurrence of relapse of malignancies. Recent studies showed a lower incidence of chronic GvHD and severe acute GvHD in patients receiving naive T cell depleted grafts compared to patients receiving complete T cell depleted grafts. On the other hand, the incidence of acute GvHD in patients receiving cord blood grafts containing only naive T cells is rather low, while potent graft-versus-leukemia (GvL) responses have been observed. These data suggest the significance of naive T cells as both drivers and regulators of allogeneic reactions. The naive T cell pool was previously thought to be a quiescent, homogenous pool of antigen-inexperienced cells. However, recent studies showed important differences in phenotype, differentiation status, location, and function within the naive T cell population. Therefore, the adequate recovery of these seemingly innocent T cells might be relevant in the imminent allogeneic reactions after allo-HCT. Here, an extensive review on naive T cells and their contribution to the development of GvHD and GvL responses after allo-HCT is provided. In addition, strategies specifically directed to stimulate adequate reconstitution of naive T cells while reducing the risk of GvHD are discussed. A better understanding of the relation between naive T cells and alloreactivity after allo-HCT could provide opportunities to improve GvHD prevention, while maintaining GvL effects to lower relapse risk.

## Introduction

Allogeneic hematopoietic cell transplantation (allo-HTC) is an intensive, but potentially curative treatment for refractory hematologic malignant disorders, and life-threatening, non-malignant diseases, such as primary immune deficiencies and inborn metabolic disease. Complications associated with this therapy, such as graft rejection, acute or chronic graft-versus-host-disease (aGvHD and cGvHD respectively), infections, and disease relapse are life-threatening and limit allo-HCT success. T cell reconstitution after allo-HCT plays an important role, as T cell numbers associate with the occurrence of relapse in malignant disease and the outcome of GvHD responses (1–7). The effect of T cell reconstitution on outcome after allo-HCT seems dependent on a delicate balance between overly active, unwanted responses to ‘self’ and building productive immunity against relapsed disease.

Transplantation with T cell depleted (TCD) grafts results in lower GvHD incidence and graft rejection, but also leads to delayed immune reconstitution (IR) and thereby increases risk of disease relapse and viral infections (2, 3, 8–11). Recently, new evidence has highlighted the importance of specifically naive T cells in GvHD and graft-versus-leukemia (GvL) responses after allo-HCT; transplantation with naive TCD grafts significantly reduced the chance of developing cGvHD and severe aGvHD (12, 13), while allowing faster IR and lower disease relapse compared to patients receiving complete TCD grafts (12–14). The knowledge about naive T cell reconstitution after allo-HCT is, however, remarkably scarce and the precise mechanisms and role for naive T cells in alloreactivity needs to be elucidated in more detail.

In this review, we will provide an overview from recent literature on the heterogeneity and composition of the naive T cell population in different graft types and discuss their role in allogeneic reactions associated with GvHD and GvL after allo-HCT. Furthermore, possible naive T cell-targeted strategies to enhance allo-HCT outcome will be suggested. More knowledge about the underlying mechanisms of naive T cells in alloreactivity after allo-HCT will allow us to gather insights in how to prevent adverse reactions such as GvHD, while maintaining GvL responses to prevent leukemia relapse.

## Naive T Cell Reconstitution After allo-HCT

Naive T cells differentiate from thymocytes in the thymus and are a heterogenous pool of T cells that have not yet encountered their cognate antigen (14–17). Thymocytes arise from hematopoietic stem cells (HSCs) in the bone marrow (BM), after which they migrate to the thymus where positive and negative selection takes place. Following transformation into a mature T cell, naive T cells migrate to the periphery and are called recent thymic emigrants (RTEs). RTEs are characterized by the surface expression of CD31, CR1 and CR2 (18), the production of IL-8, and high levels of T cell receptor excision circles (TRECs) (19). TRECs are generated during T cell receptor (TCR) gene rearrangements in the thymus, have a low intracellular degradation and do not replicate, thus can serve as a reliable marker for thymic output (20). In addition, RTEs have upregulated expression of TLR1 and appear to increase IL-8 production and upregulate CD45RO and CCR4 upon ligand stimulation (16), indicating that they are susceptible to innate stimuli. More mature naive T cells lower the production of IL-8 and express the lymphoid homing receptors CCR7 and CD62L, which are responsible for their continuous circulation between secondary lymphoid organs and the blood *via* the lymphatic system. Although the exact compartmentalization is tissue- and age-dependent, naive T cells are also widely distributed in non-lymphoid tissues, and some cells are suggested to hardly even recirculate through the blood (16, 21, 22). It is thought that tissue residency supports naive T cell maturation and long-term maintenance (22). However, the role of the tissue niche needs further investigation. Upon encounter and interaction with their cognate antigen, naive T cells will expand and differentiate into different types of effector and memory T cells in a matter of days. Recent studies on single-cell transcriptomics show that the differentiation of naïve T cells to central memory (CM), then effector memory (EM), and finally CD45RA+ EM (EMRA) T cells is accompanied by upregulation of chemokine and cytokine genes (23–26). A phenomenon also shown in innate T cells (27), and tissue resident T cells (28). Especially after HCT, single-cell sequencing of naïve T cells would provide important insight in the role of naïve T cells in GvHD and GvL. 

**Figure 1:**
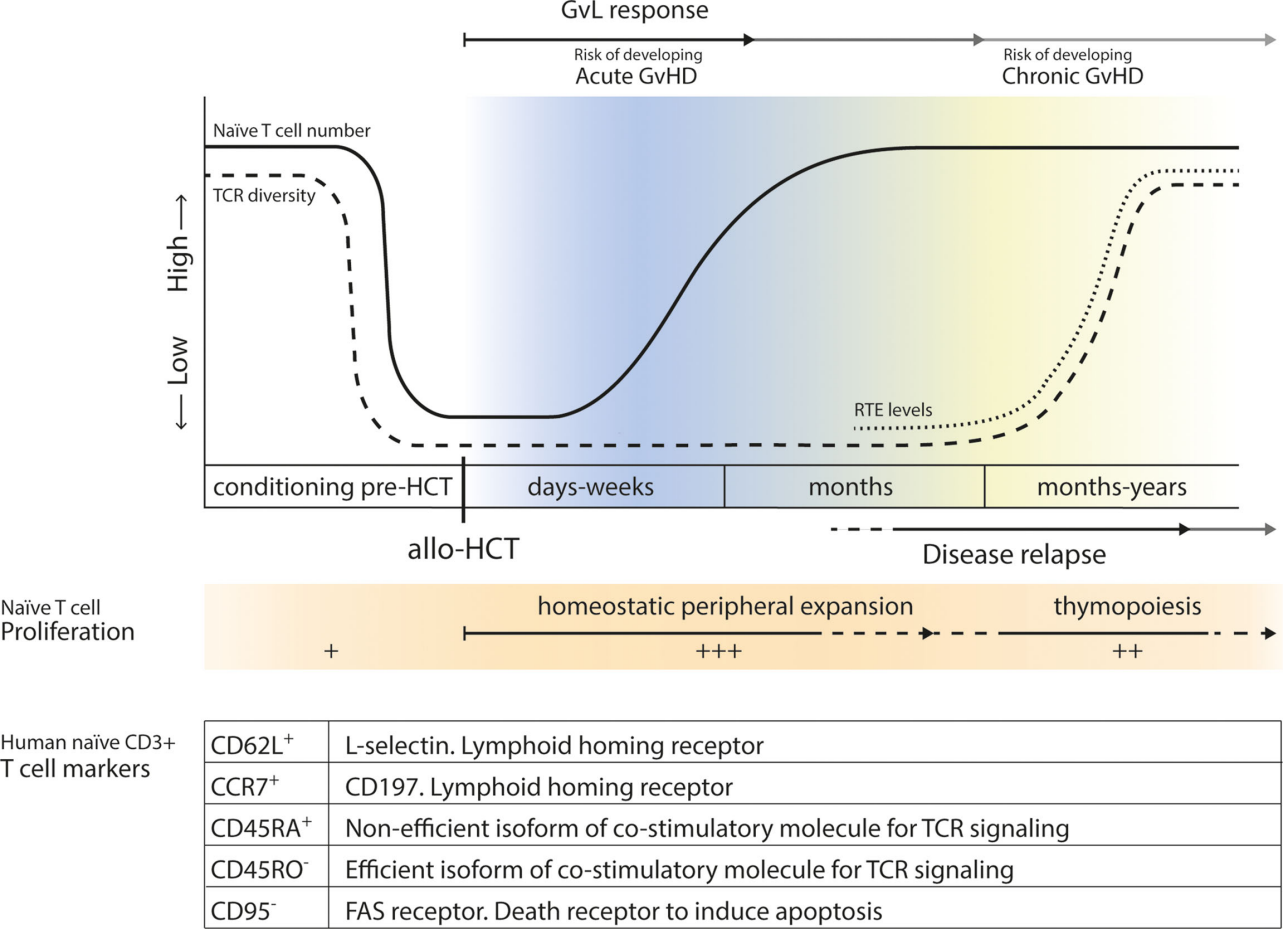
Levels of naive T cells before and after allo-HCT. Before transplantation, levels of recipient naive T cells drastically drop due to conditioning regimens, radiation and serotherapy. In the first few months after allo-HCT, after an ultimate low, lymphopenia induced HPE of donor-derived naive T cells will increase levels of naive T cells. After 6-12 months, thymopoiesis will start to be restored and production and release of RTEs leads to an increase in TCR diversity. Acute GvHD may develop early after allo-HCT, while chronic GvHD may occur later after transplantation. GvL responses contribute to the prevention of disease relapse later after allo-HCT. In the table markers associated with a naive CD3+ T cell phenotype are summarized, and their function is shortly described.

A large and diverse TCR repertoire is of great importance for the formation of an adequate immune response against newly encountered pathogens. Normally, both thymic production and homeostatic peripheral expansion (HPE) of naive T cells are responsible for the TCR repertoire diversity and size of the naive T cell pool after allo-HCT (29, 30). During the first few months after allo-HCT, the lymphopenic state of the patient results in cytokine- and antigen-driven proliferation of graft-derived (naive) T cells for compensation, which can lead to the expansion of certain T cell clones at the expense of others (31–33), significantly limiting TCR diversity. Thymic function is needed to restore TCR repertoire diversity, however, the thymus is drastically damaged due to cancer treatment and HCT-conditioning. It generally takes 6-12 months for the thymus to regenerate and restore thymopoiesis, and reconstitution to reference levels and restoration of the TCR repertoire may require years (30, 34). A limited TCR repertoire after allo-HCT has been linked to worse survival chances (35, 36), and adequate naive T cell reconstitution might, therefore, be of clinical importance after transplantation (**Figure 1**).

Early naive T cell reconstitution after allo-HCT highly depends on the number of naive T cells transferred with the graft (35, 37–39). Different types of allo-HCT grafts contain different frequencies of naive T cells; cord blood (CB) contains almost no memory T cells and CB-derived grafts, therefore, consist of a significantly larger fraction of naive CD4+ and CD8+ T cells than peripheral blood (PB-) and BM-derived grafts (40). Besides higher frequencies of naive T cells, the fast reconstitution of naive T cells after CBT might be a result of the highly proliferating fetal naive CD4+ T cells present in CB grafts (41, 42). This might explain why early naïve T cell counts are generally higher after cord blood transplantation (CBT) compared to bone marrow transplantation (BMT) and peripheral blood stem cell transplantation (PBT) (35, 37–39). In turn, PB and BM grafts contain higher frequencies of effector, central and terminally differentiated memory T cells due to the interaction with many antigens encountered during the life time of the donor (43). Notably, the neonatal naive T cell compartment differs in composition compared to the adult naive T cell compartment in BM and PB, which is extensively reviewed by van den Broek et al. (16). While naïve CB T cells recapitulate fetal ontogeny, naïve T cells after BMT resemble naïve T cells from peripheral blood (42). These fetal naive T cells in CB grafts seem to mediate a stronger anti-leukemic effect compared to adult naive T cells, and are poised to become regulatory T cells (41). The enhanced TCR signaling in CB naïve T cells is an important mechanism for the rapid CD4+ T cell restoration after CBT, as well as their strong GvL effect (41). Naïve T cells in G-CSF-mobilized PB and -BM grafts might be less functional, and more tolerogenic, since G-CSF-treatment was shown to induce immunologic tolerance and negatively affect T-cell functionality (44).

Next to early naïve T cell recovery, late recovery *via* thymopoiesis might also be different when starting with fetal- compared to adult stem cell sources. It is known that fetal and adult HSCs give rise to thymocytes with distinct gene signatures (41), indicating that thymopoiesis might be different after CBT and BMT/PBT. Indeed, the differentiation of donor-derived lymphoid progenitors in the thymus is faster after CBT compared with PBT and BMT, and a broader TCR repertoire diversity is present up to 3 years after CBT (35, 45). Thymic output increases similarly following PBT and BMT (34, 46), although higher naive T cell numbers have been reported early and later after transplantation in recipients of G-CSF-mobilized PB grafts compared with recipients of BM grafts (47). Importantly, it should be noted that thymopoiesis is significantly influenced by age, viral reactivation, immunosuppressive drugs and alloreactive donor T cells against host thymic epithelium (34, 45, 46, 48, 49).

The different naive T cell pool between graft sources, together with differences in patient characteristics, may lead to a differential naive T cell composition after allo-HCT. This possibly affects the type of immune response generated upon various types of triggers and may play a crucial role in both GvHD and GvL. Most studies show that graft-derived naive T cells are strongly related to GvHD risk (50, 51), although host tissue-resident T cells are also described to play a role (52). Naive T cells derived from thymopoiesis later after transplantation are less likely to induce alloreactivity, as they undergo selection in the patient’s thymus. Together, it would be valuable to better understand the function of naive T cells and to monitor naive T cell heterogeneity after allo-HCT.

## Naive T Cells in GvHD

GvHD is one of the major complications after allo-HCT, associated with high morbidity and mortality risks, accounting for approximately 15% of deaths (53). Acute GvHD and chronic GvHD differ in clinical presentation, time of onset after transplantation and pathogenesis (54). Incidences vary between 20-60%, depending on specific patient characteristics and treatment protocols (55–57). It is thought that naive T cells play a major role in the development of both acute and chronic GvHD (12, 58), although the exact mechanisms remain elusive.

Acute GvHD has long thought to be mediated by donor T cells, which are activated by host antigen presenting cells (APCs) in the setting of an inflammatory environment and typically occurs in the first few months after transplantation (54, 59). However, recent evidence points towards a previously overlooked role of host tissue-resident T cells. These cells are able to survive HCT-conditioning and are not only present in the skin and the gut during aGvHD but are also activated, suggested to be a result of donor APCs (52). Since transplantation with naive TCD grafts does not reduce aGvHD incidence but does reduce severity of the disease (12), suggests that both donor and host T cells may be required for severe aGvHD. Upon activation, alloreactive effector memory T cells cause tissue damage to target tissues, such as the gastrointestinal tract, liver, skin, or thymus, by direct cytotoxicity or cytokine-mediated injury (4, 60). The question remains if the effector memory T cells measured in blood of patients are directly derived from the graft and proliferate upon encounter of an alloantigen, or that these cells are differentiated from graft-derived naive T cells that recognized allo-antigens and initiated an allogeneic reaction. This difference is important and should be considered when studying the possible role of naive T cells and effector memory T cells in alloreactivity after allo-HCT.

Some studies suggest that naive T cells are even more potent in the induction of aGvHD and cause a more severe type compared to memory T cells (56, 61–63), especially because naive T cells are the most potent inducers of alloreactive responses *in vitro* (50, 51, 64). An explanation for this could be that naive T cells have a high TCR repertoire diversity and a high proliferative capacity, resulting in an increased potential of recognizing allogeneic antigens presented by host APCs and initiating alloreactive immune responses. In line with this, high quantities of CD4^+^ naive T cells in allografts correlated with a higher incidence of aGvHD after transplantation (58). Additionally, aGvHD being associated with lower counts of CD4^+^ naive T cells in the peripheral blood suggests that graft-derived naive T cells became activated in the process of aGvHD (58). Fujii et al. demonstrated that successful treatment of aGvHD by infusion of human BM mesenchymal stem cell-derived extracellular vesicles in mice was mediated by suppression of the functional differentiation of naive T cells to effector T cells (65). These results suggest that graft-derived naive T cells can recognize allogeneic antigens and subsequently induce a potent alloreactive immune response by expansion and differentiation to effector memory T cells (62–65). Notably, homing to secondary lymphoid organs might be essential for the induction of aGvHD by naive T cells, as blocking of lymphoid homing receptors or the entry of lymphoid organs almost fully abolishes aGvHD in mice (66).

In chronic GvHD, persistent tissue injury and early post-transplant inflammation due to aGvHD and conditioning can result in enhanced antigen presentation by APCs and thereby activation of allo-reactive naive T cells (67). In addition, thymic damage due to alloreactive responses in aGvHD and as a result of the conditioning are thought to be responsible for a diminished naive regulatory T cell output and a defective negative selection of auto-reactive naive T cells after allo-HCT, which may contribute to the formation of allo-reactive responses as seen in cGvHD (49, 68–71). This underlines the importance to further clarify the role of naive T cell reconstitution in GvHD. Currently, clinical trials are being conducted to further evaluate naive TCD peripheral blood stem cell grafts. Recently reported data on three phase-II clinical trials showed very low rates of cGvHD and a decreased incidence of serious aGvHD in the HLA-matched HCT setting, similar to complete TCD grafts (12, 13), with no apparent increase in relapse rates and an improved immune reconstitution compared to complete T cell depletion (12, 13, 72–74). However, it should be noted that the observed outcome in recipients of naive TCD PB stem cell grafts cannot directly be translated to other graft sources. The outcomes of PBT and BMT are rather similar, however, recipient transplanted with CB grafts already showed reduced GvHD rates compared to PBT/BMT (75, 76). Since CB grafts almost completely consist of naive T cells, this suggests the presence of phenotypic differences in the naive T cell pool between graft sources. Specifically, fetal CD4^+^ naive T cells contain the propensity to adopt a regulatory T cell phenotype upon stimulation, thereby supporting tolerance to self, and potentially foreign, antigens (41).

## Naive T Cells in GvL

The curative potential of allo-HCT therapy in leukemia patients is predominantly regulated through alloreactivity, mediated by donor T cells directed at residual malignant cells. The first study on the cure of leukemia after total body irradiation and allo-HCT was published in 1956, and important insights into underlying processes were reported in a landmark study from the International Bone Marrow Transplant Registry in 1990 (77, 78). Interestingly, they showed that usage of TCD grafts, and grafts derived from identical twins, both resulted in a reduced GvL response (78). Accordingly, it was concluded that GvL is mediated by donor T cells and is dependent on the existence of histocompatibility differences between donor and recipient (68, 78). Moreover, it was shown that both CD8^+^ and CD4^+^ T cell subsets are involved in GvL reactions by direct target killing of tumor cells due to the recognition of antigens presented by MHCI and MHCII respectively (69, 79). Outcomes of patients transplanted with naive TCD grafts confirm an important role for donor T cells in GvL; usage of complete TCD grafts resulted in increased relapse rates (13, 68, 69, 78), while the recipients of naive TCD grafts show encouraged relapse-free survival rates (12, 72–74). Moreover, acute leukemia patients with minimal residual disease receiving a haplo-HCT with ATG and G-CSF, had a significantly delayed recovery of naïve T cells compared to that of HLA-matched sibling donor recipients (80–82). However, the cumulative incidence of relapse was lower after haplo-HCT. This suggests that other lymphocyte subsets may play a role in GvL in recipients of naive TCD and haploidentical grafts.

Effector memory T cells might mediate GvL effects without causing GvHD (62, 76, 83–85). Effector memory T cells are short-lived, possess reduced cytokine production capabilities after allo-HCT, and seem to have a lower threshold for activation of GvL effects than for induction of GvH responses in murine models (83, 84). This might be due to the requirement of a sustained and high-magnitude T cell response for GvHD, and not for GvL, that effector memory T cells can not generate. Another explanation may be linked to their localization; effector memory T cells have excellent access to leukemia cells in blood, bone marrow, and spleen without expression of additional inflammatory signals (79), while GvH responses are thought to be initiated in secondary lymphoid tissue, i.e. sites that are easily accessible for naive T cells but not for effector memory T cells due to their lack of lymphoid homing receptors CD62L and CCR7 (86).

Interestingly, CBT, in which the graft almost exclusively contains naive T cells, show high GvL potential in combination with reduced relapse risk, without increased GvHD incidence compared to BMT/PBT (75, 87). CB-derived T cells exhibit exceptional learning capacity, high anti-virus properties, and powerful anti-leukemic activity (88–90). Furthermore, within two years after CBT, a higher TCR repertoire diversity can be observed compared to BMT/PBT (35, 91). The intrinsic differences of CB-derived naive T cells compared to other graft types is largely unknown and of high interest for future investigation. One possible explanation is that fetal naive CB T cells are skewed to give rise to other T cell lineages compared to adult naive T cells (41). This emphasizes the importance of in-depth phenotyping of naive T cells derived from different graft types to study their role in IR and the development of GvHD and GvL after allo-HCT. This might provide implications for the depletion of naive T cells from BM or PB grafts, leading to a reduced GvHD risk without hampering GvL responses. CB*-*derived naive T cells are more tolerogenic and unmanipulated CB grafts might thus protect against alloreactive responses and contribute to an improved immune reconstitution.

## Naive T Cell-Based Markers to Predict Allo-HCT Outcome

Several studies report a correlation of increased levels of naive T cells with the onset of cGvHD (58, 92, 93). Bohmann et al. described that both the proportion of naive cells among CD4+ T cells and absolute CD4+ naive T cell counts predict the onset of clinical symptoms of cGvHD (93). In addition, the absolute level of activated CD8^+^ effector memory T cells was shown to predict onset of aGvHD (4), showing the possible predictive value of naive T cells. However, given their lymphoid homing properties, the naive T cells present in the circulation might not be representative for the total naive T cell compartment. Future research into peripheral tissues may reveal specific niches for naive T cell maintenance and functional differentiation, which might improve understanding of the heterogenic naive T cell pool and their contribution to alloreactivity in the reconstituting immune system after allo-HCT. To achieve this, there is a need for harmonization and standardization of both the assessment methods and the markers used for naive T cell characterization. When naive T cell subsets and pool composition measurements can be compared between different transplantation centers, naive T cells can further be studied and validated as a predictive marker for GvHD.

In a study by Thus et al., a tool was developed to predict minor histocompatibility antigen mismatches and their correlation with patient survival and adverse events in different transplantation settings. Predicted Indirectly ReCognizable HLA Epitopes (PIRCHE) were used to predict donor T cell mediated recognition of mismatched-HLA derived peptides following allo-HCT, with peptides presented on HLA-I as PIRCHE-I score, and peptides presented on HLA-II as PIRCHE-II score. Those scores were correlated with patient survival, disease relapse and complication development (94). In BMT, low PIRCHE I and PIRCHE II scores in donor-recipient mismatch resembled similar allo-HCT outcomes as 10/10 matched grafts. However, in CBT, a high PIRCHE I and low PIRCHE II score in donor-recipient mismatch increased GvL responses without changing GvHD risk (94). This might lead to a new donor-recipient matching strategy in CBT to further enhance its GvL effects. Nevertheless, the mechanisms behind the different behavior of naive T cells in CBT compared to BMT and PBT remain elusive and more research into the heterogeneity and function of naive T cells is necessary to unravel this controversy.

## Naive T Cell-Based Strategies to Improve Allo-HCT Outcome

Some centers are successfully using T cell depleted grafts to reduce GvHD risk. Despite initial success, complications, such as increased disease relapse and viral reactivation have emerged, caused by delayed or absent early T cell reconstitution after allo-HCT (2). Recent efforts are focused on improving T cell reconstitution by personalized dosing, timing and duration of immunosuppressive drugs used in allo-HCT. Commonly used drugs shown to affect (naive) T cell reconstitution are serotherapy (anti-thymocyte globulin; ATG) (1, 10), fludarabine (95) and steroids (96). Importantly, significantly smaller naive T cell numbers post-HCT are present when ATG is administered prior to transplantation (97), while naive T cell transferred with the graft survive posttransplant cyclophosphamide (98). A randomized phase 2 clinical trial (Trial no. NL4836) studied the effect of ATG dosing based on weight and absolute lymphocyte counts pre-HCT. CD4+ T cell reconstitution and event free survival were improved in the individualized dosing group without affecting the incidence of graft failure and GvHD (99). Fludarabine exposure is also highly variable between patients in current dosing regimens. Optimizing fludarabine exposure in individual patients, using weight and renal function, might thus be of clinical relevance in allo-HCT (95). The pharmacokinetic/pharmacodynamic relationship of steroids in allo-HCT is currently investigated in our center (NL8703). If such a relationship exists, a clinical trial to study personalized dosing might show improved immune reconstitution and outcome after allo-HCT.

Since naive T cells show higher alloreactive capacity and are more potent inducers of GvHD compared to effector memory T cells (51, 61, 62, 83, 100–102), depleting naive T cells from BM or PB grafts (and not CB grafts) might be a strategy to prevent GvHD without delaying T cell reconstitution (103). Bleakley et al. proved the feasibility of creating naive TCD PB stem cell grafts and its safety in allo-HCT (12). Although the incidence of aGvHD was not reduced, a remarkably low rate of cGvHD (9%) was observed in patients receiving naive TCD grafts, compared to cGvHD rates after complete TCD HCT (19%) or T cell-replete HCT (40-63%) (12, 104, 105). Additionally, memory CD4^+^ and CD8^+^ T cell numbers recovered much earlier, and no significant increase in disease relapse was observed after naive TCD grafts relative to patients that received complete TCD grafts (12, 14). Furthermore, viral reactivations in patients receiving naive TCD grafts were comparable with T cell-replete grafts, and other serious infections were rare (12, 106). These results are comparable to recent data on three prospective phase-II clinical trials that showed low rates of severe aGvHD and an exceptionally low rate of cGvHD (7%), with no associations with increased relapse rates or serious infections (13). Two other phase I clinical trials also show the feasibility of memory only- or naive TCD donor lymphocyte infusions and its association with a low incidence of GvHD (72, 73). However, these studies were single-arm first in-human clinical trials, so no formal comparisons of survival were conducted. A prospective randomized controlled clinical trial is currently being performed to directly compare naive TCD PB grafts with standard unmanipulated grafts (NCT03779854). In addition, another multicenter trial focusses on the comparison of naive TCD PB grafts with complete TCD PB grafts, PBT with post-HCT cyclophosphamide and PBT with tacrolimus and methotrexate to study whether naive T cell depletion directly reduces cGvHD (NCT03970096). Nevertheless, in combination with pre-clinical data, naive TCD grafts show great potential in prevention of GvHD without delaying IR and hampering GvL responses in allo-HCT.

Thymic regeneration, thereby recovering TCR repertoire diversity (107), may take months to years, leaving patients susceptible for infections and viral reactivation with increased morbidity and mortality risk (26, 108). Strategies to enhance the restoration of thymic function after allo-HCT are therefore of high interest, as extensively reviewed by Velardi et al. (109). Exogenous administration of keratinocyte growth factor (KGF), IL-7 or IL-22 in (pre-)clinical studies has shown to be beneficial in the rejuvenation of thymic function and thereby contribute to improved thymopoiesis, enhanced naive T cell recovery, expansion of the RTE pool and increased TCR repertoire diversity (110–115). In addition, thymosin-α1 administration in allo-HCT recipients was shown to be safe in a phase I/II clinical trial and resulted in increased T cell numbers together with earlier appearance of pathogen-specific T cell responses (116). However, the success of those therapies will partly depend on the degree of damage to the thymus caused by intensity of allo-HCT conditioning and additional complications. Again, this emphasizes the importance of personalized conditioning and treatment in allo-HCT to reduce the risk of thymic damage. Moreover, when thymic function can be restored early after transplantation, *de novo* production of naive T cells will contribute to a high TCR repertoire diversity and thus high antigen specificity, thereby support a long-lasting functional immune recovery (35).

## Conclusions and Future Directions

Current insights show that the naive T cell compartment consists of a heterogenic population of T cells highly involved in GvHD and GvL responses after allo-HCT. The naive T cell pool contains the most diverse collection of TCRs, which is of great importance for the formation of adequate immune responses against newly encountered pathogens. Together with the capacity of naive T cells to promptly switch to effector phenotype and proliferate upon antigen encounter, this high TCR repertoire diversity potentiates the naive T cell pool for GvL. On the other hand, TCR diversity might be responsible for the recognition of allo-antigens and contribute to GvHD risk. Naive T cells derived from distinct graft types, such as CB versus BM and PB, show a different behavior regarding GvHD risk and GvL effect, and might thus have an intrinsic difference in the balance between GvHD and GvL alloreactivity. The differences between CB-derived and BM/PB-derived naive T cells need to be further explored and their role in alloreactivity needs to be elucidated. For this, the recent advances in tracking naïve T cells for single cell sequencing using natural barcoding, would be of major interest to better understand their role in GvHD and GvL effects (117). Moreover, factors that suppress GvHD might also limit the GvL potential of naive T cells, such as corticosteroids, conditioning with ATG, and naive T cell depletion in BMT/PBT approaches. Importantly, GvL responses by other lymphocyte populations might also be suppressed by GvHD treatment and prophylaxis. Therefore, individualized conditioning, evaluating the effect of steroid exposure, next to application of PIRCHE for donor selection, would be of interest for future study to potentiate naive T cell GvL function without exacerbating GvHD. Furthermore, harmonization and standardization of naive T cell characterization and monitoring between transplantation centers would help to further identify the role of naive T cells in GvHD development and relapse risk. More knowledge about the full composition of the naive T cell pool after allo-HCT can possibly function to better predict and understand alloreactivity. This might provide opportunities to better prevent GvHD reactions, while remaining GvL effects to lower relapse risk after allo-HCT.

## Author Contributions

LD and ES wrote the manuscript; ES designed the figure; CK and SN conceptualized and revised the manuscript; CL revised the manuscript. All authors have read and agreed to the published version of the manuscript.

## Funding

This work was supported by the Princess Máxima Center for Pediatric Oncology, Utrecht, The Netherlands.

## Conflict of Interest

The authors declare that the research was conducted in the absence of any commercial or financial relationships that could be construed as a potential conflict of interest.

## Publisher’s Note

All claims expressed in this article are solely those of the authors and do not necessarily represent those of their affiliated organizations, or those of the publisher, the editors and the reviewers. Any product that may be evaluated in this article, or claim that may be made by its manufacturer, is not guaranteed or endorsed by the publisher.
